# The Uterine Microbiota in Mares With Endometritis: Impacts of Antibiotic Treatment

**DOI:** 10.1155/vmi/5270993

**Published:** 2026-04-17

**Authors:** Gian Guido Donato, Patrizia Nebbia, Maria Cristina Stella, Fabrizia Gionechetti, Ugo Ala, Davide Cristofoli, Patrizia Robino, Alberto Pallavicini, Tiziana Nervo

**Affiliations:** ^1^ Department of Veterinary Sciences, University of Turin, Grugliasco, Italy, unito.it; ^2^ Department of Life Sciences, University of Trieste, Trieste, Italy, units.it

**Keywords:** 16S, antibiotic treatment, bacteria, endometritis, mare, microbiome, microbiota

## Abstract

Despite growing interest in the role of the reproductive microbiota, the uterine microbiota of mares has been only scarcely investigated using sequencing approaches. The aims of this study were to describe the uterine microbiota of mares with endometritis and the effects of antibiotic treatment using both 16S rRNA sequencing and culture. Five mares with clinical signs of endometritis and a positive bacteriological culture were enrolled. During the follicular phase (T0), uterine samples were collected using a double‐guarded cytobrush and swab for microbiome and bacteriological analysis, respectively. Following the antimicrobial susceptibility test, they were treated with intrauterine infusions of ceftiofur sodium, and samplings were repeated during the subsequent follicular phase (T1). According to bacterial culture, at T0, *Streptococcus equi zooepidemicus* was identified in 4 mares and *Escherichia coli* in one. At T1, 3 mares resulted negative, one was positive for *Staphylococcus xylosus*, and one continued to test positive for *E. coli.* According to NGS, the most represented genera at T0 were *Streptococcus*, *Escherichia-Shigella*, *Corynebacterium*, *Arcanobacterium*, *Porphyromonas, and Staphylococcus*. The first 3 genera dominated the microbiome of 4 mares with a relative abundance ranging from 44% to 99%. At T1, these genera’s relative abundance dropped, and the most abundant were *Acinetobacter*, *Staphylococcus*, and *Pseudomonas.* Furthermore, after intrauterine infusion of ceftiofur, the microbiome was more diverse, according to Shannon and Simpson indexes (*p* < 0.05).

## 1. Introduction

Endometritis is considered one of the major causes of subfertility in the mare [1]. It can have both infectious and noninfectious nature, and they often occur in association [2]. A transient inflammation of the endometrium is a normal response after insemination or natural mating and is necessary to clear the endometrium from excess spermatozoa, microorganisms, debris, and air [3]. Persistence of inflammation beyond 48 h after breeding indicates a predisposition to persistent breeding‐induced endometritis. This condition leads to excessive uterine inflammation that can predispose to infectious endometritis [4]. These pathogenic organisms may be latent, deep within the endometrial glands, introduced at mating, or ascend from the caudal genital tract [4–6]. Inadequate immune response, as well as impaired uterine fluid drainage and defective reproductive anatomy, are predisposing factors for infection [2].

Infectious endometritis is characterized by the presence of microorganisms in the endometrium. The most commonly isolated bacteria are *Streptococcus equi* subsp. *zooepidemicus* and *Escherichia coli* [4], but also *Pseudomonas aeruginosa*, *Staphylococcus aureus*, and *Klebsiella pneumoniae* represent etiological agents frequently isolated in equine endometritis [4].

Antibiotic administration is a recommended treatment when bacterial endometritis is diagnosed [7]. The route of administration can be systemic or intrauterine, and both are used [2, 4]. In the intrauterine administration, the effects of antibiotic treatment on the intestinal flora are low, the local concentration is higher, and the amount of antibiotic required is lower than with systemic administration [2, 4]. However, systemic treatment is also nowadays widely used because the risk of iatrogenic contamination is lower since repeated invasions of the cervix are avoided, treatment is possible regardless of the phase of the estrus cycle, and the antibiotic is evenly distributed in the uterine mucosa [4, 8].

Diagnosis of infectious endometritis is commonly based on bacterial culture [9]. However, the classical culture‐based approach can miss the great microbial diversity present in a specific environment [10] focusing only on culturable bacteria. Culture‐based techniques can only confirm the presence of microorganisms that can grow on a specific medium [11], while it is estimated that only a small fraction of microbes can be cultured in a laboratory [12, 13].

An alternative is the use of the non–culture‐based sequencing techniques targeting the 16S rRNA gene, a technique able to identify bacteria independent of cultivability [14]. Thanks to this technique, the dogma of a sterile uterus has been challenged [14, 15], and it has recently been shown that the uterus of healthy mares hosts a resident microbiota [14, 16].

In women and mammals, especially cows, specific shifts of the reproductive tract’s microbiota have been associated with fertility/infertility and uterine affections [17–27]. However, knowledge of the endometrial microbiota of the mare is still scarce and mainly focuses on the microbiota of healthy mares [14, 23, 28, 29]. Therefore, understanding more deeply the microbiota of healthy and unhealthy mares would be of great help, and it could open new frontiers in the diagnosis and treatment of uterine affections. Regarding associations between uterine microbiota and endometritis, several studies have been conducted in the woman [19, 22, 30, 31]. Furthermore, in the cow, endometritis has been associated with a lack of bacterial diversity and richness of the endometrial microbiota [18, 26]. Notably, up to date, only a few studies analyzing the microbiota of mares and jennies affected by endometritis were conducted [17, 32, 33]; however, the effects of antibiotic treatment have yet to be investigated [17, 33].

This study aimed to (a) conduct an exploratory descriptive analysis of the uterine microbiota in mares affected by endometritis, comparing the classical microbial culture with 16S sequencing; (b) evaluate the impact of the intrauterine antibiotic treatment with ceftiofur sodium on microbiota composition.

## 2. Materials and Methods

All experiments have been approved by the Ethics and Animal Welfare Committee of the Department of Veterinary Sciences of the University of Turin (Italy) (0002180, 2021). All the owners provided informed written consent, and the procedures were carried out in accordance with the EU Directive 86/609/CEE and with the guidelines of the Italian Ministry of Health for the care and use of animals (D.L. 4 March 2014 n. 26 and D.L. 27 January 1992 n. 116).

### 2.1. Animals and Experimental Design

Five broodmares with clinical signs of endometritis, a positive bacteriological culture and treated with ceftiofur sodium, were selected for this study. The mares were not experimental animals but were privately owned and were presented to two different practitioners in the province of Turin (Italy) for insemination. The choice of drug therapy was carried out by the two practitioners. The sampling was performed during 2022 and 2023 breeding seasons. The included mares met the following criteria: three or more consecutive unsuccessful natural mating/artificial insemination attempts using fertile semen; clinical signs of endometritis (excessive uterine edema and intrauterine fluid accumulation > 2 cm [1]); positive for bacterial culture; and no antibiotic exposure in the preceding 3 months.

The mares were aged 5–19 years old. All the mares foaled at least once, and, when the study was performed, they were barren. One mare was an Arabian, 1 was a Standardbred, and 3 were Thoroughbreds. The mares were housed in shared paddocks, fed with hay, and pellet feed, and water was supplied ad libitum. The mares were housed on three different farms situated in the province of Turin, Northern Italy.

During the follicular phase (T0), determined as the presence of a follicle > 25 mm and uterine edema (score ≥ 2/5 [34]), uterine samples were collected using a double‐guarded cytobrush for microbiota analysis, immediately followed by a double‐guarded swab for bacteriological analysis.

Antibacterial susceptibility tests (AST) were performed, and according to the AST result, the five mares were treated with intrauterine infusions of ceftiofur sodium (1 g daily for 3 days, Wondercef, FATRO, Italy). The day following each infusion, a uterine lavage with sterile saline solution was performed until the efflux was clear.

A second round of sampling was conducted during the subsequent follicular phase (range 18–26 days, average 21.6 ± 3 days after the first sampling) (T1).

### 2.2. Sample Collection

The perineum was washed three times with water and povidone–iodine soap (Betadine; MEDA PHARMA SpA, Milan, Italy) and then dried with a paper towel.

Wearing a sterile glove, a double‐guarded uterine cytobrush and a double‐guarded swab (Minitube GmbH, Tiefenbach, Germany) were used to collect a sample for bacteriological culture and microbiota analysis avoiding contaminations from the vagina. Briefly, the instrument was introduced in the vagina and moved to the internal cervical os, where the inner tube was pushed out; once the tip reached the uterine body, also the brush/swab was pushed out. The instrument tip was rolled against the endometrium for 30 s and retracted into the outer tube before removal of either the cytobrush or the swab from the uterus.

Subsequently, the tip of the brush was cut using sterile scissors and stored into a sterile 2‐mL Eppendorf tube (Sigma‐Aldrich, St. Louis, USA) and kept refrigerated at 4°C until reaching the laboratory, with a time lag of 2 h maximum. The samples were frozen without any prior processing in sterile Eppendorf tubes and stored at −80° until further processing.

The swabs were placed in AMIES transport medium (Minitube GmbH, Tiefenbach, Germany) and transported, within 2 h at refrigeration temperature, as above, to the Microbiology Laboratory of OVU for processing.

### 2.3. Bacterial Analysis

Bacterial cultures were performed according to established protocols, as previously described [35]. Briefly, each uterine swab was resuspended in 3 mL of a nonselective enrichment medium (Brain Heart Infusion Broth Oxoid) at 37°C overnight; then, each sample was streaked on agar with 5% sheep blood plates for a further 24 h at 37°C in a 5% CO_2_‐enriched atmosphere. Bacterial isolates were identified using MALDI‐TOF/MS and the software MALDI‐Biotyper (Bruker, Germany), subjected to AST (disk diffusion method), and performed and interpreted according to the Clinical and Laboratory Standards Institute (CLSI) M27‐A3 (2018) and EUCAST guidelines.

### 2.4. 16S rRNA Gene Sequencing

DNA extraction and sequencing were carried out using established protocols as previously described [36]. DNA extraction from the frozen and thawed brushes was performed using the E. Z. N. A Soil DNA Kit (Omega Bio‐Tech). Laboratory reagents were used as negative controls to check for possible contamination by the extraction kit. In addition, two clean sterile brushes were used as negative controls to check for possible contamination from the swabs. DNA was quantified using the NanoDrop 2000 spectrophotometer (Thermo Fisher Scientific). The hypervariable region of 16S rRNA V1‐V2 was used because it significantly reduces off‐target amplification of host mitochondrial DNA at low microbial biomass due to its lower similarity to the mammalian mitochondrial genome [36, 37]. Primers V1‐V2 were tailed with i5 and i7 adapters from NextEra to enable barcoding in a second amplification step. PCR was performed in a 25 μL volume reaction containing 12.5 μL AccuStart II PCR ToughMix 2X (Quanta Bio), 1.25 μL EvaGreen 20X (Biotium), 1 μL 16S‐i5‐XT‐27F primer (10 μM), 1 μL 16S‐i7‐XT‐338R primer (10 μM), and 2 μL (50 ng) DNA template. PCR was performed in a CFX 96 PCR System (Bio‐Rad) with a real‐time limited number of cycles of 94°C for 30 s, 55°C for 20 s, 72°C for 30 s, and a final extension of 72°C for 5 min. All amplicons were checked for quality and size by electrophoresis of 2 μL on 2% agarose gel and visualization of *a* ≈ 350 bp band. Amplicons were then sent to an external laboratory (BMR Genomics, Padua, Italy) for barcoding and sequencing using the Illumina MiSeq platform (Illumina, San Diego, CA).

### 2.5. Data Analysis

Sequencing data were initially processed and analyzed using CLC Microbial Genomics Module Version 23.0.3. Raw reads were quality‐filtered to remove adapters and low‐quality sequences with a quality score threshold of 0.03. Paired‐end reads were merged, and primers were trimmed. Paired‐end reads were denoised into amplicon sequence variants (ASVs) using the DADA2 algorithm [38] implemented in the CLC Microbial Genomics software. The resulting ASVs were taxonomically classified using the SILVA database Version 138.1 with a confidence threshold of 97%. All sequences of nonbacterial origin (i.e., chloroplasts and sequences not assigned to the Bacteria kingdom) were removed.

Data analyses were further conducted using *R* ver. 4.2.2 (Vienna, Austria) using the packages phyloseq, vegan, ggplot2, microbiome, and DESeq2. Data were rarefied based on minimum library size. Alpha diversity was calculated by Observed species, Chao 1, Shannon, and Simpson indexes using a pairwise Wilcoxon test; beta diversity was estimated using Bray–Curtis analysis. Permutational multivariate analyses of variance (PERMANOVA) were run based on Bray–Curtis dissimilarity to assess differences after the treatment. Differential abundance was calculated at the genus level using DESeq2. Significance was considered for *p* < 0.05.

## 3. Results

### 3.1. Bacterial Analysis

After bacterial culture, *S. equi* subsp. *zooepidemicus* was identified in four mares, while *E. coli* was isolated in one mare. At T1, 3 mares resulted negative to culture. However, mare 3 showed a positive result for *Staphylococcus xylosus* and mare 1 continued to test positive for *E. coli* (Table 1).

**TABLE 1:** Bacterial uterine culture result before and after antibiotic treatment, age, and parity.

Mare *n*°	Before antibiotic treatment (T0)	After antibiotic treatment (T1)	Age (years)	Parity (*n*)
1	*Streptococcus equi zooepidemicus*	—	7	2
2	*S. equi zooepidemicus*	—	19	5
3	*S. equi zooepidemicus*	*Staphylococcus xylosus*	5	1
4	*Escherichia coli*	*E. coli*	7	2
5	*S. equi zooepidemicus*	—	6	1

*Note:* The results of AST are reported in supporting materials (S1).

### 3.2. 16S rRNA Sequencing

A total of 922,028 pair‐end reads were obtained. After the filtering step, 790,350 reads remained resulting in 4124 ASVs. Rarefaction curves reported in supporting materials (S2) show that the sequencing depth was sufficient to observe the total diversity present in the microbial community.

In total, bacteria belonging to 18 phyla were observed. Specifically, 14 phyla were identified at T0, while 18 were identified at T1. At the genus level, 299 different genera were identified. Among these, 92 were detected at both T0 and T1, while 34 were present specifically at T0 and 173 at T1 (S3).

**FIGURE 1:**
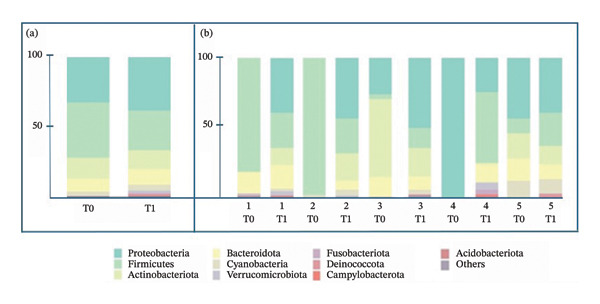
(a): Relative abundance (expressed in percentage) of the 10 most abundant phyla detected at T0 and T1, grouped according to time point. (b): Relative abundance (expressed in percentage) of the 10 most abundant phyla detected before (T0) and after (T1) treatment in each mare.

Firmicutes was the most abundant phylum at T0, with a relative abundance of 38.7%. Proteobacteria was the second most abundant phylum (34.1%), followed by Actinobacteriota (14.8%), Bacteroidota (8.9%), and Cyanobacteria (2.9%). At T1, the most abundant phylum was Proteobacteria (39.6%), followed by Firmicutes (27.7%), Actinobacteriota (13.2%), Bacteroidota (11.1%), and Cyanobacteria (4.1%). These phyla accounted for more than 95% of the total relative abundance, while the remaining phyla showed a relative abundance < 1.5%. Relative abundance at the phylum level is shown in Figure 1. Relative abundances of all the 18 phyla for each mare and grouped per time point are shown in the supporting materials (S4 and S5, respectively).

At T1, the relative abundance of the genus *Streptococcus* dropped to 0.7%, and the same was observed for *Escherichia-Shigella* (1.9%) and *Corynebacterium* (3.1%). The most abundant genera at T1 were *Acinetobacter* (5.4%), *Staphylococcus* (4.9%), and *Pseudomonas* (4.6%), whose relative abundance, on the contrary, increased after the antibiotic treatment. Furthermore, these genera were followed by *Roseomonas* (4.2%), UCG‐004 (2.7%), *Sarcina* (2.6%), *Rikenellaceae* RC9 (2.4%), *Clostridium* (2.3%), and *Sphingomonas* (2.3%).

**FIGURE 2:**
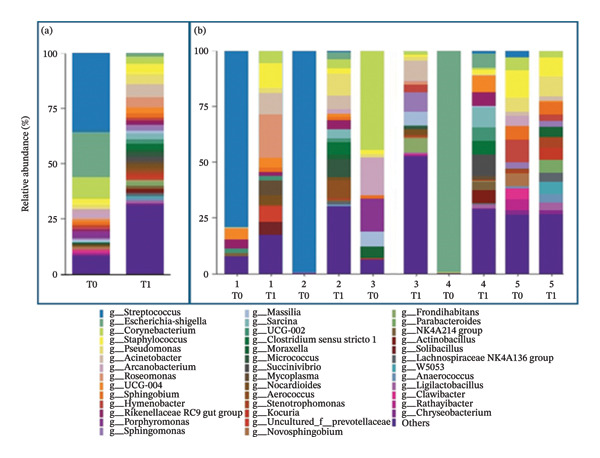
(a): Relative abundance (expressed in percentage) of the 40 most abundant genera detected before (T0) and after (T1) treatment grouped according to time point. (b): Relative abundance (expressed in percentage) of the 40 most abundant genera detected before (T0) and after (T1) treatment in each mare.

Mares 1 and 2, which tested positive for *S. equi* subsp. zooepidemicus by bacterial culture, showed a relative abundance of the genus *Streptococcus* of 79% and 99% at T0, respectively. Following treatment, the relative abundance decreased to 0% and 1.1% at T1. Mare number 4, which was positive for *E. coli*, had a relative abundance of 99% of the genus *Escherichia-Shigella* at T0. At T1, the relative abundance decreased to 6.4%. Mare numbers 3 and 5, also positive for *S. equi zooepidemicus*, showed a modest relative abundance of *Streptococcus* of 0.3% and 3.4% at T0. However, in mare number 3, the genus *Corynebacterium* represented 44.4% of the abundance at T0 and decreased to 1.4% at T1. Relative abundance at the genus level is shown in Figure 2. Relative abundances of the 40 most abundant genera for each mare and grouped per time point are shown in the supporting materials (S6 and S7, respectively).

### 3.3. Alpha Diversity, Beta Diversity, and Differential Abundance

**FIGURE 3:**
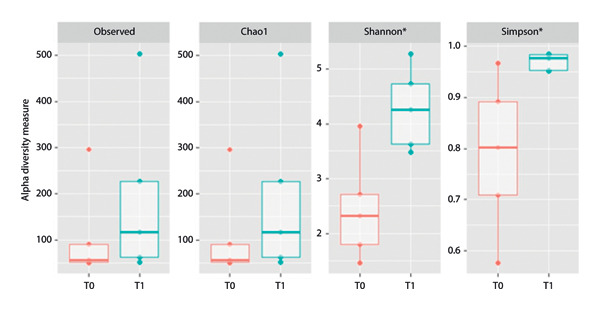
Variation in endometrial microbial diversity based on time point (before antibiotic treatment—T0, after antibiotic treatment—T1) assessed by Observed richness, Chao1, Shannon, and Simpson. ^∗^ indicates a *p*‐value < 0.05.

An increase in alpha diversity was observed after the antibiotic treatment, as described through Shannon (*p* = 0.032) and Simpson indexes (*p* = 0.032). For Observed species and Chao 1 indexes, the *p*‐value was 0.35 (Figure 3).

**FIGURE 4:**
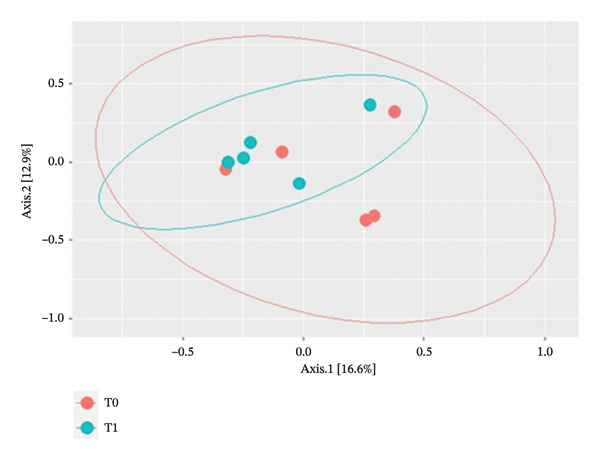
Two‐dimensional plot by Bray–Curtis based on time points (T0 and T1). The first two axes explain 29.5% of the total variation.

PCoA based on Bray–Curtis dissimilarity showed substantial overlap between samples collected at T0 and T1, with no clear separation by time point. Accordingly, beta diversity did not differ significantly between the two time points (*p* = 0.6) (Figure 4).

Differential abundance analysis performed with DESeq2 identified the genera *Roseomonas* and *Mycoplasma* as significantly more abundant at T1, whereas *Streptococcus* was more abundant at T0 (S8).

## 4. Discussion

This paper describes the endometrial microbiota of a relatively small group of mares with endometritis using both bacterial culture and 16S sequencing. Furthermore, the effects of antibiotic treatment were also investigated, as the role of therapies for uterine infections on the uterine microbiome has not been studied yet in horses [2]. It is important to highlight and critically discuss the limitations of this study. One limitation is the small number of animals included. Another limitation is the absence of cytological examination. However, although it could have influenced the initial classification of the status of the mare, this does not undermine the relevance of the findings regarding the impact of antibiotic treatment on the uterine microbiota or the comparison between bacterial culture and 16S rRNA sequencing.

Recently, the endometrial microbiota of the healthy mare has been investigated [14, 28, 39]. Consistent with our findings, previous studies have reported Firmicutes, Bacteroidetes, Proteobacteria, and Actinobacteria as the most abundant phyla in the equine uterus [14, 28, 29, 39]. Virendra et al. [33] reported that in healthy mares, Firmicutes predominated over Proteobacteria, whereas the opposite pattern was observed in mares with endometritis. In contrast, our findings showed Firmicutes as the dominant phylum in affected mares, with Proteobacteria surpassing them after antibiotic treatment.

At the genus level, Holyoak et al. [14] described a core uterine microbiota of healthy mares composed of *Lactobacillus, Escherichia-Shigella, Streptococcus, Blautia, Staphylococcus, Klebsiella, Acinetobacter,* and *Peptoanaerobacter.* Furthermore, according to another study by Heil et al., the dominant genera were *Klebsiella*, *Mycoplasma*, and *Aeromonas* [28]. The present work shows that the two most abundant genera at T0 were *Streptococcus* and *Escherichia-Shigella*, confirming the results of bacterial culture that isolated bacteria belonging to these genera (namely, *S. equi zooepidemicus* and *E. coli*) in the 5 mares. These bacteria are indeed two of the etiological agents most frequently isolated in equine endometritis [4, 35, 40]. Furthermore, *Corynebacterium* was the third most abundant genus followed by *Arcanobacterium, Porphyromonas, Staphylococcus, Hymenobacter, Pseudomonas*, *and Sphingobium.* Accordingly, in mares with endometritis, Virendra et al. [33] reported *Escherichia—*followed by *Salmonella* and *Klebsiella—*as the predominant genus, thereby reinforcing the involvement of *Escherichia* in the pathogenesis of the disease. Conversely, Guo and colleagues [32] identified *Burkholderia* and *Chlamydia* as the most abundant genera in the endometrial microbiota of both healthy and affected mares, with a significantly higher relative abundance observed in mares with endometritis. These discrepancies among uterine microbiota studies underscore the challenges of comparing findings across research conducted in different geographic locations and using varying sampling methods and enrollment criteria. Large‐scale studies are therefore needed to overcome these limitations and generate results that are comparable and clinically applicable.

After the treatment, the relative abundance of the genera *Streptococcus*, *Escherichia-Shigella*, and *Corynebacterium* decreased. This decrease in the abundance of bacteria that were isolated by cultural examination and that dominated the microbiota at T0 shows the effectiveness against these bacteria of targeted antibiotic treatment performed according to the antimicrobial sensitivity test. In contrast, an increase in *Pseudomonas, Staphylococcus*, and *Acinetobacter* was observed. The increase in the relative abundance of *Pseudomonas* could be attributed to the documented low activity of ceftiofur on species belonging to this genus: In a recent paper by Pottier et al., resistance to ceftiofur was observed in 98% of *Pseudomonas aeruginosa* strains isolated mainly from equine reproductive tract [41]. However, the overall relative abundance at T1 was 4.6%, a value not higher than 27% observed in healthy mares according to Holyoak et al. [14]. Furthermore, the relative abundance of *Staphylococcus* and *Acinetobacter* was 4.9% and 5.2%, respectively, and these genera have been described as residents of the core microbiota of healthy mares [14]. According to the differential abundance analysis, the genus *Streptococcus* was significantly more abundant before treatment (T0), whereas the genera *Mycoplasma* and *Roseomonas* were significantly more abundant after treatment (T1). The higher abundance of *Streptococcus* at T0 compared to T1 is consistent with the presence of endometritis and the subsequent administration of antibiotics; however, the role of *Mycoplasma* and *Roseomonas* at T1 remains unclear, and it is not yet known whether their increased abundance reflects a positive or negative shift in the post‐treatment uterine microbiota. Furthermore, the limited sample size necessitates cautious interpretation of these differential abundance findings.

In this study, mares treated with an intrauterine infusion of ceftiofur sodium were enrolled. This antibiotic is a third‐generation cephalosporin and is commonly used to treat endometritis [42, 43]. According to current recommendations [44], it should only be used in the presence of clinical signs, positive cultures, and antimicrobial susceptibility testing [4]. The choice of the antibiotic was performed by the two practitioners responsible for the insemination of the mares. Even if all the isolated bacteria were sensitive to ceftiofur and the mares presented clinical signs, probably other first‐line antimicrobials could have been used [44].

Investigating the overall diversity described through alpha diversity measures, we showed that after the antibiotic treatment, the microbiota is more diverse, according to Shannon and Simpson indexes, two measures of diversity that account not only for the number of species observed but also the relative abundance of each species. At T0, there is a dominance of one or few species that account for most of the abundance in the environment. At T1, on the other side, there are more species, and they are more equally distributed. Therefore, according to previous studies [14, 45], it was shown that even bacterial genera that are commonly considered uterine pathogens are normal residents of the microbiota of healthy mares, such as *Streptococcus*, *Staphylococcus*, *Escherichia-Shigella*, *Pseudomonas*, and *Corynebacterium*. However, we observed that in mares with endometritis, these bacteria dominate the uterine microbiota, which is therefore in a state of dysbiosis.

Recent studies in mares have highlighted a clear association between reduced bacterial diversity and uterine disease. Guo et al. [32] reported a significant reduction in microbial diversity in mares with endometritis compared to healthy controls, indicating the presence of dysbiosis. Similarly, dysbiosis characterized by the dominance of a single genus has been described in nocardioform placentitis: In 77% of samples from affected mares, a single genus (*Amycolatopsis*, *Crossiella, Lentzea, Mycobacterium*, or *Enterococcus*) accounted for more than 70% of the relative abundance, and the Shannon diversity index was lower compared to healthy mares [46]. These findings in horses are consistent with studies in cattle [47]. In this species, although the specific composition of a healthy uterine microbiota remains debated, bacterial diversity is considered essential for maintaining uterine health. Endometritis and metritis have been associated with diversity reduction, characteristic of dysbiosis, and an increased relative abundance of intrauterine pathogens [18, 20, 26, 47, 48].

Regarding the effect of antibiotic therapy on uterine microbiota, we observed an increase in overall bacterial diversity after ceftiofur administration and a decrease in the abundance of uterine pathogens that dominated the microbiota at T0 (*Streptococcus* and *Escherichia-Shigella*). To our knowledge, the impact of antibiotic treatment on the uterine microbiota has not previously been documented in mares. However, our findings align with studies in cows: Jeon et al. described that a single subcutaneous injection of ceftiofur crystalline‐free acid in animals with metritis resulted in an increase in uterine bacterial diversity and a reduction of *Fusobacterium*, a known uterine pathogen [49]. In another study on cows with metritis treated intramuscularly with ceftiofur, ampicillin trihydrate, or untreated, success in curing metritis was associated with an increase in the diversity of the uterine microbiota and a decrease in the relative abundance of pathogenic bacteria, such as *Bacteroides*, *Porphyromonas*, and *Fusobacterium* [50].

Interestingly, beta diversity did not differ between the two groups, and the samples did not form separated clusters based on time point (T0 and T1). Probably, this finding could be explained because the biological variability between the 5 mares enrolled was higher than the differences between the two groups based on time points. It has been shown that geographic location strongly influences the uterine microbial composition, and samples from mares from different locations form different clusters based on geographical origin [14]. In our study, the mares came from three different breeding farms, and two different pathogens were isolated in culture. The high variability and the small number of animals enrolled have probably influenced the beta diversity result.

Comparing the results of bacterial culture and 16S, in most of the mares we saw a substantial agreement, as the bacteria isolated in culture were the two most abundant bacterial genera according to metagenomic sequencing. However, in two mares positive for *S. zooepidemicus*, the relative abundance of the genus *Streptococcus* was low and not different from the one observed in healthy mares in other studies [14]. On the contrary, a high relative abundance of *Corynebacterium* was detected with 16S but not with culture technique. This result could be explained by the differences in techniques, as sequencing can also identify dead or fragmented microbial DNA and thus possibly detect dormant or biofilm‐producing bacteria. On the contrary, dead and fragmented DNA does not replicate in the female reproductive tract and is therefore not detected by traditional culture methods [28]. Another explanation could lie in the sampling technique, as only a small segment of the uterus is sampled with uterine swabs or brushes, and therefore, focal infections may have been missed by one or the other sample, leading to different results between the two techniques [2, 51]. A solution could be represented by sampling with low‐volume lavage, which, although used in previous studies [14, 28], is more difficult to perform, time‐consuming, and susceptible to contamination if specific double‐guarded systems are not used. However, according to a recent study, no significant differences in alpha or beta diversity were observed between the two techniques [28].

## 5. Conclusions

In conclusion, we demonstrated that in mares with endometritis, the genera *Streptococcus* and *Escherichia-Shigella* dominate the endometrial microbiota, which is characterized by reduced diversity. The treatment with intrauterine infusions of ceftiofur sodium determined a decrease in the relative abundance of these genera and an increase in bacterial diversity. Moreover, we compared bacterial culture and NGS sequencing in a clinical context, and we used the two techniques to monitor the effects of antibiotic treatment in mares affected by endometritis.

## Author Contributions

G.G.D. conceptualization, investigation, formal analysis, and writing–original draft. P.N. conceptualization, investigation, and writing–review and editing. F.G. conceptualization, methodology, investigation, and writing–review and editing. M.C.S. conceptualization, investigation, and writing–review and editing. P.R. conceptualization, investigation, and writing–review and editing. U.A. formal analysis and writing–review and editing. D.C. investigation. A.P. conceptualization, methodology, investigation, supervision, and writing–review and editing. T.N. conceptualization, methodology, supervision, and writing–review and editing.

## Funding

This research did not receive any specific grant from funding agencies in the public, commercial, or not‐for‐profit sectors.

Open access publishing facilitated by Universita degli Studi di Torino, as part of the Wiley ‐ CRUI‐CARE agreement.

## Conflicts of Interest

The authors declare no conflicts of interest.

## Supporting Information

Additional supporting information can be found online in the Supporting Information section.

## Supporting information


**Supporting Information 1** S1: Results of Antimicrobial sensitivity test.

**Supporting Information 2** S2: Rarefaction curves.

**Supporting Information 3** S3: Total number of Phyla and Genera at T0 and T1.

**Supporting Information 4** S4: Relative abundances of all the 18 phyla for each mare at T0 and T1.

**Supporting Information 5** S5: Relative abundances of all the 18 phyla grouped per time point (T0 and T1).

**Supporting Information 6** S6: Relative abundances of the 40 most abundant genera for each mare at T0 and T1.

**Supporting Information 7** S7: Relative abundances of the 40 most abundant genera grouped per time point (T0 and T1).

**Supporting Information 8** S8: Significantly different genera between T0 and T1 according to DESeq2.

## Data Availability

The data that support the findings of this study are available from the corresponding author upon reasonable request.
