# Supplementary material for: The Uterine Microbiota in Mares With Endometritis: Impacts of Antibiotic Treatment
Source: Vet Med Int. 2026 Apr 17;2026:5270993. doi: 10.1155/vmi/5270993 (PMC13090575; doi:10.1155/vmi/5270993)
Supplement: Supplementary file 1 — Supporting Information 1 S1: Results of Antimicrobial sensitivity test. [file VMI-2026-5270993-s007.docx]

*S1: Results of antimicrobial susceptibility testing (disk diffusion test) on tested bacterial strains.*

| Strains |  |  |  |  |  |  |  |  |  |
| --- | --- | --- | --- | --- | --- | --- | --- | --- | --- |
|  | AK | AMP | CTF | GM | P | ENO | AMX | MAR | SXT |
| S. equi mare #1 T0 | nt | S | S | nt | S | R | nt | R | R |
| S. equi mare #2 T0 | R | S | S | nt | nt | R | nt | R | nt |
| S. equi mare #3 T0 | S | nt | S | nt | S | S | S | S | S |
| *E. coli* mare #4 T0 | R | S | S | S | nt | S | S | S | S |
| *S. equi* mare #5 T0 | nt | S | S | nt | S | S | nt | S | S |
| *S. xylosus mare* #3 T1 | S | S | S | S | R | S | R | S | S |
| *E. coli mare* #4 T1 | S | R | S | S | nt | S | nt | S | R |

*Abbreviations: AK: amikacin; AMP: ampicillin; CTF: ceftiofur; GM: gentamicin; P: penicillin; ENO: enrofloxacin; AMX: amoxicillin; MAR: marbofloxacine; SXT: trimethoprim-sulphonamide;*

*S: Susceptible; R: Resistant; nt: not tested.*
