# Supplementary figures and images for: The Uterine Microbiota in Mares With Endometritis: Impacts of Antibiotic Treatment
Source: Vet Med Int. 2026 Apr 17;2026:5270993. doi: 10.1155/vmi/5270993 (PMC13090575; doi:10.1155/vmi/5270993)

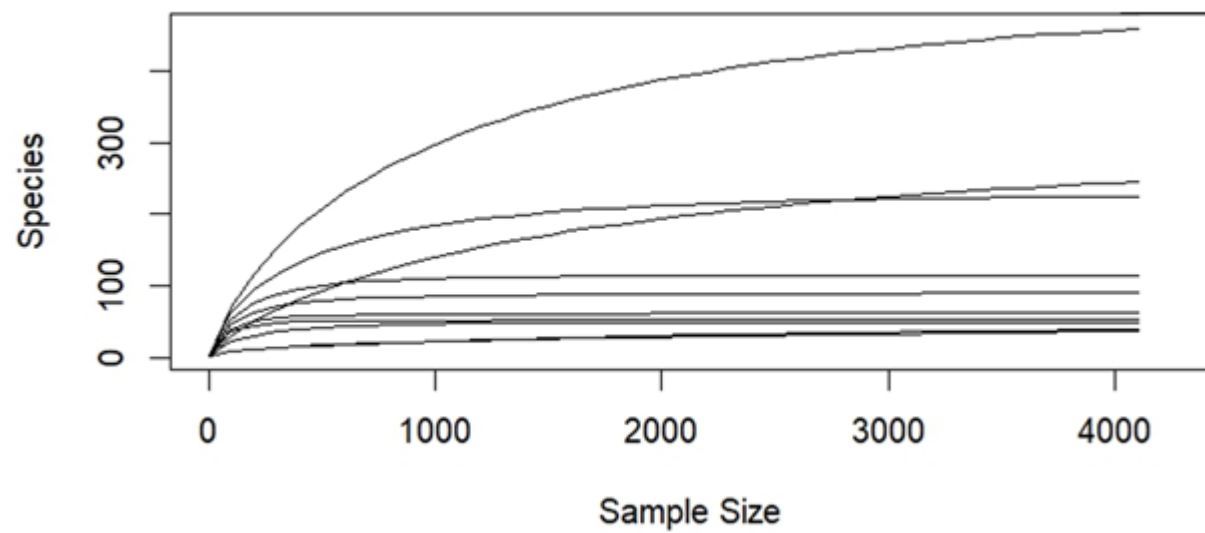

Supplement: Supplementary file 2 — Supporting Information 2 S2: Rarefaction curves. [file VMI-2026-5270993-s001.pdf]

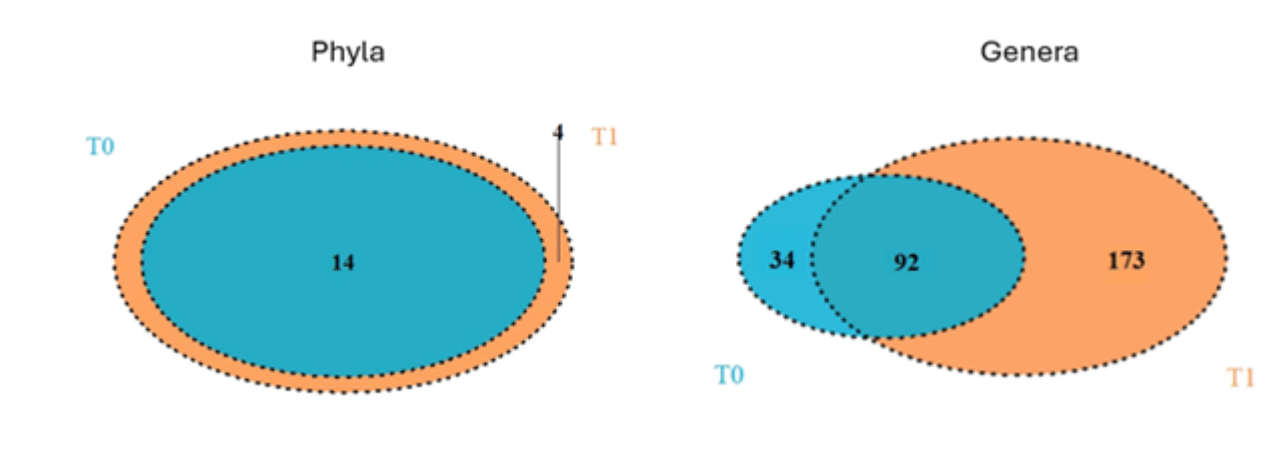

Supplement: Supplementary file 3 — Supporting Information 3 S3: Total number of Phyla and Genera at T0 and T1. [file VMI-2026-5270993-s002.pdf]
